# The plausibility transition model for sensemaking

**DOI:** 10.3389/fpsyg.2023.1160132

**Published:** 2023-05-26

**Authors:** Gary Klein, Mohammadreza Jalaeian, Robert R. Hoffman, Shane T. Mueller

**Affiliations:** ^1^MacroCognition, LLC, Arlington, VA, United States; ^2^Cognitive Systems Engineering Laboratory, Department of Integrated Systems Engineering, College of Engineering, The Ohio State University, Columbus, OH, United States; ^3^Institute for Human and Machine Cognition, Florida, IL, United States; ^4^Michigan Technological University, Houghton, MI, United States

**Keywords:** plausibility, transitions, sensemaking, stories, expectancies, surprises

## Abstract

When people make plausibility judgments about an assertion, an event, or a piece of evidence, they are gauging whether it makes sense that the event could transpire as it did. Therefore, we can treat plausibility judgments as a part of sensemaking. In this paper, we review the research literature, presenting the different ways that plausibility has been defined and measured. Then we describe the naturalistic research that allowed us to model how plausibility judgments are engaged during the sensemaking process. The model is based on an analysis of 23 cases in which people tried to make sense of complex situations. The model describes the user’s attempts to construct a narrative as a state transition string, relying on plausibility judgments for each transition point. The model has implications for measurement and for training.

## Introduction

Considerable experimentation has been conducted on how people make plausibility judgments in tasks situated in the laboratory. In contrast, we conducted a naturalistic study based on actual cases of plausibility judgments concerning complex events, as opposed to laboratory experimentation. We define plausibility judgments in the context of story-building, whereas much of the experimental literature describes plausibility judgments in terms of features, outliers, and calculation of the number of scenario versions that can be recalled or constructed.

Our thesis is that when people make plausibility judgments about an assertion, an event, or a piece of evidence, they are gauging whether it makes sense. We can therefore treat plausibility judgments as sensemaking activities, relying on story-building. We define sensemaking as the effort to connect our experiences in terms of the primary causes that we believe are operating. Our interest is in the plausibility of causal relationships represented through narrative, as opposed to broader issues such as the plausibility of maps and so forth.

Plausibility judgments are important because they come into play in a variety of ways such as comprehension, problem solving, and anomaly detection, as well as sensemaking. Connell and Keane (2004) stated that, “Plausibility has the hallmarks of a phenomenon so pervasive and important that, like air, no one notices it. Time and again, many cognitive accounts appeal to the idea of plausibility without specifying its cognitive basis” (p. 185). In his writings on abductive inference, Charles S. Peirce asserted that the formation of a novel hypothesis leads to a judgment of plausibility to determine what constitutes a “best” explanation (Hoffman et al., 2022).

Weick (1995) described the nature of sensemaking in organizations. The very first sentence in his book is “Sensemaking is tested to the extreme when people encounter an event whose occurrence is so implausible that they hesitate to report it for fear they will not be believed.” (p. 1) Weick listed plausibility as one of the seven properties of sensemaking–that sensemaking is driven by plausibility rather than accuracy.

Our research effort is an attempt to unpack the nature of plausibility judgments, which is bound up in causal reasoning and explanations.

Hoffman et al. (2011) reviewed the literature on the criteria for what counts as a cause for an effect and identified three factors: co-variance (the putative cause comes before the effect), mutability (the putative cause was theoretically reversible), and propensity (the putative cause had the potential to bring about the effect). Hoffman et al. (2011) relabeled “propensity” as “plausibility” because “propensity” puts the focus on the putative cause whereas “plausibility” puts the focus on the person.

**Figure 1:**
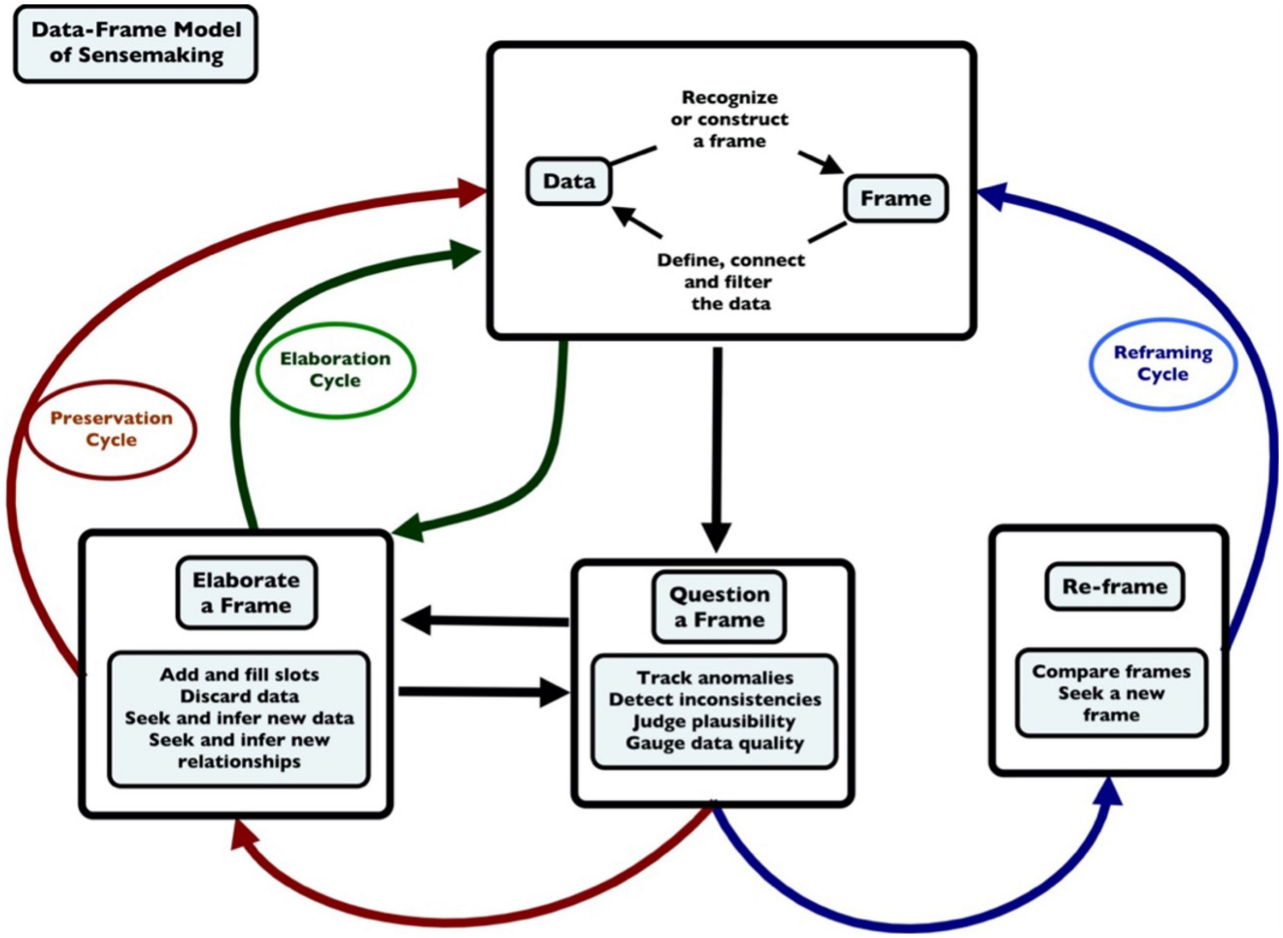
The data/frame model of sensemaking (adapted from Klein et al., 2007).

The Data/Frame model of sensemaking (Klein et al., 2006a, 2007), posits that the sensemaking process is typically initiated when someone questions an existing frame. This questioning involves tracking anomalies, detecting inconsistencies, judging plausibility, and gauging data quality. The plausibility transition model, which will be described later in this article, was clearly influenced by the Data/Frame model, including framing, questioning of the frame, and elaborating the frame (Figure 1).

We now believe that plausibility judgments are relevant for elaborating a frame, the left-hand side of the diagram, as people judge whether the elaboration is acceptable, and also for the right-hand side of the diagram, as people engage in the Re-Framing activity, specifically, “seeking a new frame.”

## Literature review

We conducted a literature review in order to learn more about how plausibility judgments are made. We wanted to examine different kinds of research projects investigating plausibility judgments, and to identify some existing theoretical accounts of plausibility judgments.

The core of our literature review was formulated by searching several databases (Academic Search Complete, Annual Review of Psychology, and Google Scholar) using the Boolean phrases Plausibility Judgment OR Plausibility Reasoning OR Plausibility Gap. This search yielded 1,042 articles, of which we judged that 18 were strongly relevant to our project. We also added four more articles that had not surfaced in the initial search but were identified as releavant by outside reviewers. We grouped this set of 22 articles, some theoretical and some empirical, into four categories: Philosophical/Logical/Analytical, Computationall/Information Processing, Linguistic, and Cognitive/Sensemaking. These categories were not mutually exclusive. Further, the range of synonyms (e.g., probability, possibility, reasonableness, feasibility) makes a comprehensive search unmanageable. Therefore, our search must be seen as a sampling of the way plausibility judgments are addressed in four different fields, and there may well be other research perspectives we have not identified thus far.

We describe the general methods and findings of these four categories in the next subsections.

### Philosophical/logical/analytical perspective

In a theoretical paper, Leake (1995) distinguished several types of plausibility criteria: a minimality criteria consistent with Occam’s razor, a proof-based criterion, and criteria that are based on costs and probabilities. Leake (1995) presented a model for understanding everyday abductive reasoning, which is the process of forming a plausible explanation for an observed phenomenon based on past experience and current goals. Leake (1995) argued that this type of reasoning is guided by a combination of experience, goals, and context, and that it plays a crucial role in our ability to navigate the world and make decisions. The model suggests that everyday abductive explanation involves a cycle of observation, hypothesis formation, testing, and refinement, which allows us to generate and evaluate multiple explanations until a satisfactory one is found.

Isberner and Kern-Isberner (2016) were interested in developing a formal approach to plausibility for knowledge representation. They conducted an experiment in which plausibility was manipulated by presenting text that was either logical or that violated logic. For example, a plausible item was *“Frank has a broken pipe. He calls the plumber”* and an implausible item was “*Frank has a broken leg. He calls the plumber.”* They measured the delays in response time as a function of the match or mismatch between background world knowledge and the text statements. The intention of the experiment was to provide evidence that one’s plausibility monitoring is in fact a routine component of language comprehension. They found that there is a delay in reactions when an implausible word was presented, and a positive response was required. Isberner and Kern-Isberner (2016) concluded that the delays in response time were caused by belief revision processes. Such processes are necessary to overcome the mismatch between plausible context and implausible target items.

Lombardi et al. (2016b) hypothesized that information is judged as more plausible if it is less complex and requires less conjecture. They studied the relationship between evaluation, plausibility, and the refutation text effect–the phenomenon where exposure to a refuting text (i.e., a text that contradicts a previous belief) can lead to stronger beliefs in the refuted idea. The authors examined different models that aim to explain this effect, focusing on the role of evaluation (e.g., attitudes, emotions) and plausibility (i.e., the degree to which an idea seems believable). Lombardi et al. (2016b) showed the connection between evaluation, plausibility, and the refutation text effect.

In a theoretical article, Lombardi et al. (2016a) defined plausibility as a function of the trustworthiness and quality of the source material. They explored the role of plausibility judgments in conceptual change and epistemic cognition. They argued that people’s understanding of the world is constantly evolving as they encounter new information, and that this process is guided by the judgments they make about the plausibility of different ideas and explanations. The paper described how these judgments are influenced by factors such as prior knowledge, experience, and goals, and how they can play a role in the selection and rejection of explanations. Lombardi et al. (2016a) also discussed how these judgments can change over time as people gain new information, and how this can lead to conceptual change.

### Computational/information processing perspective

Collins (1978) offered a computational approach that treats plausibility as a function of the certainty of the information and the certainty of the inferences. Collins (1978) explained how people arrive at conclusions that seem reasonable, even if they may not be strictly accurate. Collins (1978) suggested that people use mental shortcuts and biases to quickly form opinions and make decisions, relying on intuitive judgments and past experiences rather than rigorous analysis. The theory also highlighted the role of emotions and social influence in shaping our reasoning. The aim was to provide a comprehensive understanding of human reasoning and how it affects our decision-making processes.

In an empirical study, Collins and Michalski (1981) contrasted different types of possible inferences: argument-based, reference-based, descriptor-based, and term-based. They showed how to develop mathematical models and algorithms that capture the ways in which people reason and make decisions. This research drew on a range of fields, including cognitive psychology, artificial intelligence, and philosophy. It sought to understand the cognitive processes underlying human reasoning and to develop computational models that could simulate these processes.

Abendroth and Richter (2020) presented an information processing account of plausibility. They considered the importance of plausibility judgments in identifying fake news–information on social media that is accidentally or, most commonly, deliberately false. The study suggested that when individuals are presented with a plausible explanation for an unfamiliar scientific phenomenon, their comprehension of the topic improves. Abendroth and Richter (2020) argued that people are more likely to accept and retain information that is presented in a plausible manner, as it aligns with their existing knowledge and beliefs. The results highlighted the importance of making scientific information accessible and plausible for the public to enhance their understanding and engagement with science.

Lombardi et al. (2016a) described plausibility as “what is perceived to be potentially truthful when evaluating explanations,” (p. 35). They proposed a computational model for explaining how people determine the plausibility of events and explanations. The model suggested that people use their prior knowledge and expectations to form beliefs about the likelihood of events, and that these beliefs are influenced by multiple factors, including the coherence of the explanation and the level of detail provided. The model also proposed that people make a trade-off between the desirability of the outcome and the likelihood of the event, with more desirable outcomes being seen as more plausible. The authors argued that their model provides a framework for understanding how people make decisions based on plausibility.

### Linguistics perspective

Plausibility has been addressed in the field of linguistics as a unique cognitive process and separate from other central issues in cognitive psychology. Connell (2004) explored the factors that influence people’s judgments of the plausibility of explanations using two experiments. The study found that two key factors, concept coherence and distributional word coherence, play a significant role in shaping people’s beliefs about the plausibility of an explanation. Concept coherence refers to the degree to which an explanation is consistent with people’s existing knowledge and beliefs, while distributional word coherence refers to the similarity of words used in the explanation to words used in other related contexts.

Connell and Keane (2004) argued that explanations that are both conceptually coherent and have high distributional word coherence are more likely to be seen as plausible. The results of their study suggested that these two factors can have a significant impact on people’s judgments of the credibility of explanations. The study highlighted that the way a particular scenario is described can influence the amount of prior knowledge activation. If little background knowledge is activated, it would be difficult to understand the scenario and if a lot of background knowledge is activated, then the comprehension of the scenario would be easy. Thus, two factors can affect the plausibility judgment of a scenario: the word-coherence of the description and the concept-coherence of the scenario’s elements and events (Connell and Keane, 2004). The word-coherence (scenario word choice) factor, according to Connell and Keane (2004), is associated with the comprehension stage and more specifically the amount of time one would need to make a plausibility judgment. However, the concept-coherence (simple or complex scenarios) factor can be influential in both stages of plausibility judgment including comprehension (time) and assessment (level of plausibility a scenario). Word-coherence eases the understanding of a scenario by activating prior knowledge and concept-coherence eases the understanding and determines assessment accuracy by the amount of prior knowledge activation. For instance, when someone is asked to assess the credibility of the statement “The bottle rolled off the shelf and smashed on the floor,” they might infer that the rolling caused the smashing. This explanation may seem plausible to them due to their past experiences with fragile objects breaking when they fall and hit the floor. This explanation has a certain level of consistency with their prior knowledge and beliefs. On the other hand, if the statement was “The bottle rolled off the shelf and melted on the floor,” they could still make a causal connection, but it seems less credible as they have limited past experiences of fragile objects melting upon hitting the floor. A scenario could be created where this could happen, such as if the room was made of metal and heated like an oven, but this explanation lacks consistency with their prior knowledge and beliefs.

In another experimental study, Traxler and Pickering (1996) conducted eye-tracking experiments to investigate the effect of plausibility/implausibility on sentence comprehension. They explored the relationship between plausibility and the processing of unbounded dependencies in language. The study used eye-tracking to measure people’s processing of sentences with unbounded dependencies (sentences where the relationship between the subject and the verb is not immediately clear) to see how their assessment of plausibility influenced their processing. The results showed that people’s judgments of the plausibility of a sentence influenced the way they processed unbounded dependencies, with more plausible sentences being processed more efficiently. The authors argue that these findings have important implications for understanding how people process and comprehend complex language and how the notion of plausibility influences the interpretation of language.

Matsuki et al. (2011) explored how plausibility affects language comprehension in real-time. The study found that people make judgments about the plausibility of events as they are reading, and that these judgments have a direct impact on their comprehension of the text. The results suggested that people use their prior knowledge and expectations to form beliefs about the likelihood of events, and that these beliefs guide their understanding of the text.

All three of these perspectives (Philosophical/Analytical, Computational/Information Processing, and Linguistic) are interesting and useful. Most of them seem fairly consistent with each other. And we have no blanket disagreement with any of the plausibility criteria suggested by these researchers–logical consistency, credibility of sources, consistency with other information consistency among explanatory causes or concepts, reduced complexity, perception of truthfulness, alignment with prior beliefs, knowledge and understanding, probability, coherence, comprehensibility, ease of recalling similar instances, physical mechanism, and so forth.

However, we did not see a reflection of research and modeling that involved sensemaking. In all of the papers we reviewed in these three categories, only one mentioned causal inference–Lombardi et al. (2016a) raised the issue of physical mechanisms, which seemed to imply causality–but that was only a brief and passing mention. Connell and Keane (2004) alluded to causal factors in their discussion of prior knowledge as they studied the way subjects made plausibility judgments of textual material–sentence pairs.

The fourth category, the Cognitive/Sensemaking Perspective, was more closely aligned with the field of Naturalistic Decision Making.

### Cognitive/sensemaking perspective

Cognitive researchers have not specifically specified plausibility judgments the way the other three communities discussed above have, but have examined a number of topics that bear directly on plausibility judgments.

Although Matsuki et al. (2011) defined plausibility as the acceptability or likelihood of a situation or a sentence describing it, Connell and Keane (2006) defined plausibility as the degree of fit between a given scenario and prior knowledge.

Lombardi et al. (2016a) described plausibility as “What is perceived to be potentially truthful when evaluating explanations,”

Sinatra and Lombardi (2020) argued that people detect fake news by assessing the credibility of the source and appraising lines of evidence, along with comparisons to alternatives and probabilistic reasoning. For Sinatra and Lombardi (2020), plausibility judgments are essential for identifying fake news by relying on individuals’ subjective perception of potential truthfulness of statements. How well does an item of information conform with a reader’s prior knowledge, beliefs, or current understanding of a situation? They argued that people detect fake news by assessing the credibility of the source and appraising lines of evidence, along with comparisons to alternatives and probabilistic reasoning. The article explained that in the post-truth era, where people are often bombarded with misinformation and false claims, it’s crucial to reappraise the way we evaluate sources of scientific evidence and claims. Sinatra and Lombardi (2020) suggest that people’s judgment of what is plausible is often influenced by factors such as emotions, biases, and previous experiences. This can lead to acceptance of false information as truth and undermine the credibility of scientific evidence.

Lombardi et al. (2016a) wanted to facilitate the conceptual change on the part of students, particularly on topics such as climate change and evolution, for which large numbers of students appeared to hold views that are discrepant with scientific findings. They defined plausibility as judging the potential truthfulness of statements and concepts and made a connection between plausibility and other concepts such as probability, coherence, comprehensibility, credibility, and believability.

Lombardi et al. (2016a) also raised the topic of individual differences in need for cognition and openness to conceptual change. And they emphasized the distinction between “cold cognition” which emphasizes information processing issues such as knowledge structures, and logical reasoning, and “warm cognition” that also involves affect and motivation. Their Plausibility Judgment in Conceptual Change (PJCC) model has implications for the understanding of plausibility judgments and the use of these judgments for helping students and laypeople revise their mental models in the direction of current scientific thinking.

Lombardi et al. (2016a) drew on the work of Kahneman and Klein (2009) in claiming that plausibility judgments can be “automatic” and intuitive (System 1) as well as deliberative and analytical (System 2). Further, the authors argued that analogies can be useful in gauging plausibility.

Along this line, Nahari et al. (2010) suggested that instances are judged more plausible if it is easier to recall similar instances, essentially the availability heuristic identified by Tversky and Kahneman (1974). Nahari et al. (2010) explored the factors that influence people’s judgment of the credibility of narratives. The study found that people’s assessment of the credibility of a narrative is influenced by several factors, including the language used, the plausibility of the events described, and the degree of absorption the listener experiences while hearing the story. Nahari et al. (2010) argued that language plays an important role in shaping people’s beliefs about the credibility of a narrative, with certain linguistic cues and patterns being associated with higher credibility. Additionally, the more plausible the events described in a story, the more likely people are to believe it. Finally, the level of absorption a person experiences while hearing a story has a strong impact on their credibility judgment, with highly absorbing stories being seen as more credible. The article highlighted the importance of considering these factors when evaluating the credibility of narratives.

Connell (2004) and Connell and Keane (2004) proposed a Knowledge-Fitting Theory which identifies two stages of the plausibility judgment process, a comprehension stage (understanding the scenario) and an assessment stage (examining scenario fit to prior knowledge). To make a plausible judgment, people try to create a mental link between what the scenario describes and the previous knowledge they have about the scenario. The core of the KFT is the strength of relationship between the scenario and prior knowledge.

Some additional articles addressed sensemaking but without mentioning plausibility judgments. We see their work as quite related to our investigation of plausibility judgments.

Maguire et al. (2011) and Foster and Keane (2015) described the judgment of surprisingness in terms of sensemaking difficulty. The sensemaking accounts of surprise and explanation form the basis of our description of plausibility judgments, as discussed in subsequent sections. However, with the exception of the work by Klein et al. (2006a,b, 2007), none of the other sensemaking investigations adopted a naturalistic perspective–they relied on a laboratory paradigm, and tightly constrained stimuli such as short passages crafted to increase or decrease plausibility. Here is one of the stimulus sets used by Foster and Keane (2015), the Rebecca at the Beach scenario.

Setting: Rebecca is on the beach. She goes for a swim in the water.

Known (plausible) continuation: after she dries herself off she notices that her skin has turned red.

Less-known (less plausible) continuation: after she dries herself off she notices that her skin has turned turquoise.

### Assessment

Some of the papers we studied did mention anecdotes involving real-world settings, but none used such anecdotes or incident accounts as data to be analyzed. Therefore, we saw an opportunity to add to the existing research and models by conducting a research project examining actual incidents with complex plots and contextual implications. We studied textual accounts of explanations that we found in books, magazines, newspapers, and social media–cases in which people created written documents in an attempt to explain events and systems to readers.

## Understanding the process of forming explanations

Klein et al. (2021) investigated the process of forming explanations–the ways that people react when they encounter unexpected, anomalous, and surprising items of information and how they try to diagnose what happened. We collected a corpus of 73 cases that were a convenience sample. We did not formulate criteria in advance because we wanted to cast a wide net that did not systematically exclude any types of incidents.

For this current project on plausibility, we identified a subset of 23 cases of explanations taken from Klein et al. (2021). The criteria for this subset included having richer details about the explainer or learner’s reasoning; and coming from sources we judged to be more reliable than our initial sample. Otherwise, the 23 cases should also be considered to be a sample of convenience because we did not use content or topics to pre-establish criteria for the inclusion or exclusion of cases inasmuch as this was an exploratory study and we did not want to pre-judge the issues. The subset of 23 cases is listed in Appendix A, and includes the Air France 447 crash, the USS Vincennes shootdown, the workings of AlphaGo, and the grounding of the cruise ship *Royal Majesty*. Most of the 23 cases involved events that had taken place but several involved general accounts of systems, such as #4 How does AlphaGo work? We consider this effort to be an initial exploration of what can be learned by taking a naturalistic perspective for examining plausibility judgments.

The method we used was to review the details of each of these selected cases. The cases were examined by a single reviewer, the senior author. He tried to imagine himself as the protagonist in the incident who was seeking to gain an understanding, using the information available at the time. The reviewer then examined the reports of each of the cases and relying on induction and abduction synthesized these accounts to formulate a general model of the sensemaking involved and how it depended on plausibilityjudgments.

## The plausibility transition model

**Figure 2:**
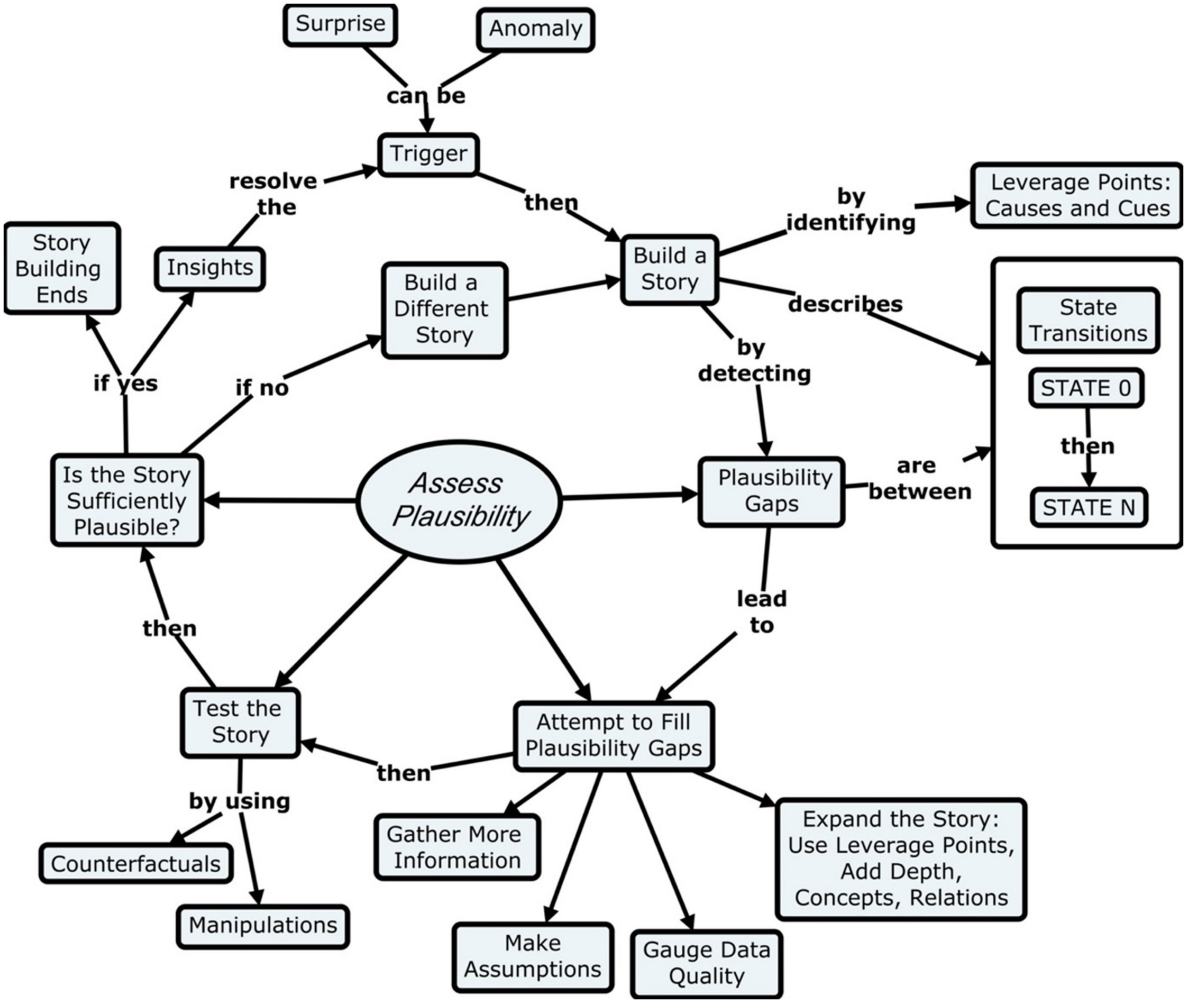
The process of story-building.

What people try to do when explaining an anomaly or surprise is to construct a story about how something came to pass or a story about how a device works. But this notion that we are building a story is banal. What matters the most is the process of story-building that people go through. This process is detailed in Figure 2, which is one of the primary contributions of our research effort. In building a story, the reasoner works out a causal sequence, going from the beginning state (State-0) to the end state (State-N), shown in the right-hand side of Figure 2. In addition, a good story will also contain an insight—the resolution of a surprise. If we are going to the trouble of building a story then there must be a reason—something we do not understand, or something that surprised us.

Figure 2 presents the model as a cycle (a closed loop), beginning with the trigger that initiated the story-building process. This trigger is usually a surprise or a desire to overcome ignorance. The loop closes when the plausibility assessment is judged to be sufficient, and the insights gained resolve the triggering conditions. If people determine that the story is not adequately plausible, they try to build a different story and start the process again. Figure 2 emphasizes the function of *Assessing plausibility* during the story building process. It is the force driving the explanatory process, and is shown in the center as an oval, not a box because it is not a stage in the process. It is included in the figure to show which of the stages are involved in assessing plausibility.

### Surprise: triggering the explanation process

Figure 2 shows that the explanation process of story-building is triggered by surprises and anomalies. Cognitive researchers have explored the process of surprise. Kahneman and Miller (1986) posited norm theory as a rapid means for gauging typicality and anomaly. Norms are constructed *ad hoc* by recruiting specific representations and exemplars. The normality of a stimulus is evaluated by comparing it to the norms it evokes, using the heuristics of availability and simulation. Foster and Keane (2015) and Munnich et al. (2019) advanced a sensemaking account of surprise as a metacognitive estimate of the effort needed to explain an anomaly. Reisenzein et al. (2019) provided an alternative description of surprise, not as the effort involved but in terms of discrepancies and prediction failure. See also Chinn and Samarapungavan (2008) and Chinn (2017) for discussions of the process of surprise and conceptual change.

### Plausibility judgments

One of the primary forces that guides story-building is plausibility. For each state transition, from State-0 to State-N, the reasoner gages the plausibility of that transition.

Our notion of plausibility is psychological, not philosophical, linguistic or computational. Plausibility judgments entail plausibility gaps that need to be filled in. These plausibility gaps stimulate curiosity. People search for additional information that will fill in the gaps.

In addition, plausibility judgments operate at a second level shown in Figure 2–assessing the efforts to fill the plausibility gaps within the state transitions. People try to fill the gaps by making assumptions–a key part of the explaining process. If the plausibility gaps cannot be satisfactorily filled, confidence in the story is reduced or even lost. People may shift into a “snap-back” mode, as described by Cohen et al. (1997). During an incident, if the initial diagnosis is wrong, the anomalies will persist and increase. So, the effort of explaining away the anomalies and filling the gaps becomes greater and greater, either because there are more and more anomalies to explain away, or because the effort at explaining away is just too great. At that point, people become skeptical of the story and start searching for an alternative story, or they become more receptive to an alternative story that they had been dismissing. Conversely, when it is easy to build the mental simulation of how the state transitions work, people tend to judge those transitions as plausible. This is discussed in the judgment and decision-making literature as the simulation heuristic (Kahneman and Tversky, 1984).

To make plausibility judgments for the transitions in a story, we hypothesize that a person engages in mental simulation, imagining each state transition as a response to the causes of which the person is aware. If a state transition flows smoothly, then there is no problem. On the other hand, if the known causes do not account for the transition, then people experience this mismatch as a strain, the cognitive strain described by Cohen et al. (1997). If the strain becomes too great, some sort of mental “tilt” arises, and people lose faith in the story. Figure 2 shows how people might try to reduce the cognitive strain by gathering more information, assessing the quality of the anomalous data (and hoping to find a reason to disregard the anomalous data), making assumptions, or by expanding the story. However, at its core, this psychological judgment of plausibility hinges on imagination. Even in imagining a physical process, say a panel operator for a petrochemical plant, the operator will be imagining, “if I were a molecule of ethane in this splitter unit of a petrochemical plant, and I was exposed to this level of heat, how much pressure would I be feeling? Will my passage up the distillation tower be expedited? And if so, how quickly will this reaction take, once a set of control actions are taken?”

Our account is consistent with Kahneman and Miller’s (1986) presentation on norm theory and the recruitment of exemplars to judge typicality.

### Leverage point identification

To identify leverage points for building a story a person has to draw on knowledge of the types of causes for events such as those that triggered the explanation process. This causal set is activated just-in-time in response to surprise or to ignorance. In the course of self-explaining, the causal set will be expanded and deepened, and a person’s mental model will become richer–the overall causal repertoire will be expanded. The leverage points people identify (the causes considered, and the cues noticed) will depend on the sophistication of the person’s mental model and the kinds of stories the person has considered in the past. So, stories determine the leverage points that are identified, and the leverage points identified will activate a set of causes and make certain cues more salient.

### The process of deepening

When do people deepen their story? Rozenblit and Keil (2002) discussed the illusion of explanatory depth, suggesting that people do not deepen enough—that people are satisfied with a shallow understanding. But surely people cannot deepen all the way down because there is no “all the way down.” There are always additional questions to examine and additional possibilities for unpacking the concepts being introduced. So, we stop deepening when we are satisfied with the plausibility of the story. Therefore, the Plausibility Transition model also describes a stopping rule for ceasing to expand and deepen a story—when the plausibility is experienced as sufficient.

Story-building continues as a person introduces leverage points and clues into the causal chain and network until the person has an account that satisfies his/her sense of plausibility: “Yes, this makes sense, it could easily have happened this way.” One of the 23 cases we studied is the analysis presented by Gladwell (2014) about David Koresh and the Waco Texas tragedy. The behavior of the Branch Davidians seemed completely irrational at the start, but by the end of Gladwell’s account, their behavior made a lot of sense. It was plausible.

Our account is consistent with Foster and Keane’s (2015) discussion of bridging inferences that fill in the plausibility gaps. Foster and Keane speculate that the effort in creating these bridging inferences is based on the difficulty of retrieving the appropriate information, formulating the inferences, and integrating the information. These cognitive operations all seem relevant and raise the question of whether there is anything more to judging plausibility. It may be that retrieval and integration are sufficient to account for the cognitive strain we have postulated. However, a story-building account goes beyond retrieval and integration. It is not simply the difficulty of the retrieval and integration (see also Costello and Keane, 2000, on conceptual combination), but the phenomenological experience of having the pieces of the story click into place. Here we are dealing with a causal account (Hoffman et al., 2011). Further, the difficulty is experienced as an emotional reaction–the “warm cognition” feature that Lombardi et al. (2016a) emphasized.

### Causal repertoires

What counts as causes for accidents, anomalies, and surprises? We generally know the types of things that come to mind as potential causes. These include events, decisions, forces, missing data, erroneous data, and flawed beliefs. But these factors are too general. For the analysis of the 23 cases of story-building, we found that we were attending to potential causes even as we were constructing the story. The plausibility transition model assumes that people have causal repertoires–their mental models include a capability for generating potential causes for a given outcome. Different people would have different causal repertoires of the types of things they would consider in building a story. What does the plausibility transition model look like when applied to actual instances? We use an example, the Air France Flight 447 accident, to answer this question.

### Air France flight 447

The Airbus 330 of Air France airlines took off from Rio de Janeiro in Brazil on June 1, 2009, headed to Paris, carrying 228 passengers and crew members. Several hours into the flight, ice crystals formed on the pitot tubes, preventing the airplane from determining its speed. As a result, the autopilot turned off. The airplane was using the latest in intelligent technology and the manufacturers had led aircrews to believe that it was impossible to stall the plane. The airplane was just too smart and would not allow pilots to engage in unsafe actions that might result in a stall. And that was true as long as the sensors were working. However, with the autopilot disengaged, the intelligent safeguards no longer operated. Now the airplane could enter into a stall. Unfortunately, the pilot flying, the most junior member of the aircrew, apparently did not know this (or had never been told it).^1^ So he continued to climb steeply, feeling a false sense of invulnerability. The plane was in fact climbing so steeply, and its airspeed was so reduced, that it was on a trajectory to stall.

At some point, the pitot tubes seem to have unfrozen, even though the autopilot did not come back on. Now the airplane did sense the airspeed and did identify the near-stall condition. As a result, a stall warning came on. This auditory warning confused the pilot flying who thought the airplane was installable. He continued climbing. And then the stall warning went off. The stall warning ceased due to the slow speed (presumably because you do not want the stall warning going off while the airplane is taxiing on the ground). The pilot flying must have felt relieved that the stall warning went off and took this as a good sign instead of a very ominous sign.

**Figure 3:**
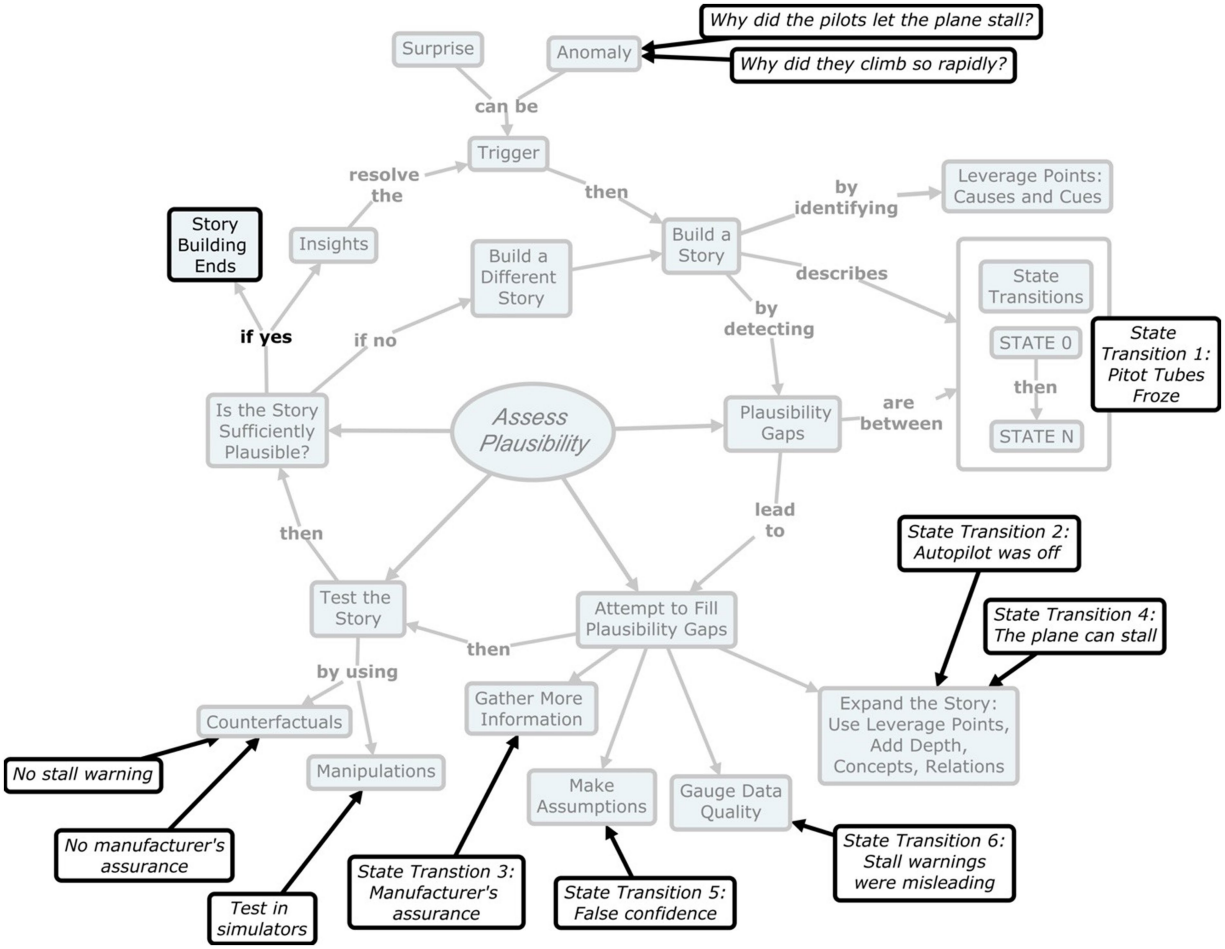
Applying plausibility gap model judgment in air France flight 447 example.

The more experienced first officer realized the flight configuration was extremely dangerous. He seems to have taken over the controls and he put the nose down to increase the speed. As a result–the stall warnings came back on. This happened because the airspeed had increased over the minimum. Now the pilots were thoroughly befuddled. Putting the nose down (to escape the stall conditions) was getting them yelled at by the system but continuing to climb was absurd. As they tried to sort out what was happening, the airplane did stall, and it dropped into the ocean. It was several years before the airplane was located and the flight data recorder could be recovered. No one had imagined that a jetliner could be flying so slowly. As a result, the stall warning, intended to help the pilots avert danger, actually helped to kill them. Figure 3 maps these events onto the plausibility transition model.

## Relation of the plausibility transition model to other models

Existing accounts of plausibility judgments seem incomplete: they emphasize logical analyses, scrutiny of the credibility of sources, and so forth, all of which are important, but fail to consider plausibility judgments as a form of sensemaking.

Our thesis is that there may be value in taking a sensemaking perspective on plausibility judgments. Gauging that something is plausible or implausible is, at some level, assessing whether or not it makes sense to the individual, and even to the team.

The plausibility transition model in some ways resembles the knowledge-fitting theory of plausibility (Connell and Keane, 2004). Their work did highlight the role of prior knowledge, especially causal knowledge, in making plausibility judgments. They studied the assessment of textual materials, and how participants in their experiments made what they are told fit what they knew about the world. They were addressing what we refer to as sensemaking.

Our account differs from theirs in a few important ways. First, we were considering natural events, such as the crash of Air France 447, rather than textual stimuli such as “The bottle fell off the shelf. The bottle smashed” vs. “The bottle fell off the shelf. The bottle melted.” Second, our account centers around story-building and state transitions as opposed to overall plausibility judgments of statements. Third, and most critical, our account hinges on the psychological strain of imagining the state transitions whereas Connell and Keane calculated plausibility in terms of the number of scenario versions that could be recalled or constructed given the way the stimulus material was primed.

The number of scenario versions is a proxy for the cognitive strain of imagining a transition but seems fundamentally different. For example, one of our examples, the USS Vincennes shootdown of an Iranian airliner in 1988, depended on the Vincennes captain’s efforts to explain away data that were inconsistent with each of the two stories he was comparing: the object his crew had identified was a commercial airliner or it was a military airplane preparing to attack his ship. There was more cognitive strain to explain away the inconsistencies in the commercial airliner story, and so he rejected that story (Klein, 1998). This type of plausibility judgment is easy to handle by the Plausibility Transition account of Figure 2. In contrast, the Connell and Keane account is not really designed to describe judgments of this kind.

Our account is most closely aligned with the PJCC (Plausibility Judgments in Conceptual Change) model of Lombardi et al. (2016a). Their emphasis was on scientific beliefs, whereas ours is on the construction of stories to account for events. However, we did include several cases centered on beliefs rather than events, and we did not see any differences in the plausibility transition model for these cases.

## Conclusion

The plausibility transition model has a number of important features:

- Focus on human cognition. This is different than a focus on logical analyses or source credibility.
- Story-building process. Our approach views plausibility judgments as the attempt to construct a story, a narrative, to explain the phenomenon of interest.
- Limitations of story-building. Stories generally have a sequential form, a chain of causes, which is often a necessary simplification but sometimes an over-simplification that misses explanations involving multiple intersecting causes.
- Anomaly detection is not simply noticing outliers, although that is how it is often treated. We detect anomalies when our expectancies are violated. Therefore, statistical analysis of outliers is a misleading and insufficient treatment of anomaly detection. Our views here align with those of Foster and Keane (2015), Maguire et al. (2011), and Lombardi et al. (2016a).
- State transitions. The sequential structure of stories can be seen as state transitions, moving from one state to the next as new events and information are received and as the causal implications are worked out. These state transitions can be considered as a form of mental simulation (Klein and Crandall, 1995).
- Filling gaps. Typically, the state transitions will leave gaps–the causes present in one state do not neatly align with the following state.
- Cognitive strain. These gaps pose problems for assessing the story as plausible versus implausible. The cognitive strain makes it more difficult to accept a story as plausible. This feature aligns with the concept of bridging inferences (Foster and Keane, 2015).
- Tactics for filling the gap and resolving implausibility are to gather more information, to determine flaws in the data collection process that identified the gap, to make assumptions, and to elaborate the story (adding depth, additional concepts and relationships) in order to explain away the gap.
- Centrality of plausibility judgments to story-building. Plausibility judgments are embedded in the process of constructing stories to account for surprises and anomalies.
- Double dose of plausibility judgments. Plausibility judgments are embedded in story-building in two ways: in assessing the state transitions within the story, and in the activity of modifying the story in response to plausibility gaps.
- Ease of imagining the state transitions. This ease of imagining, which is related to the availability heuristic identified by Tversky and Kahneman (1974) is a subjective and emotional judgment that the causal factors in the precursor state are sufficient to account for the subsequent state. This judgment depends heavily on the sophistication of the person’s mental model of causes.
- Existence of a stopping point. The sensemaking process that utilizes plausibility judgments does not go on forever. The stopping point occurs when the plausibility gap is reduced, and cognitive strain is minimized. This aspect of the Plausibility Transition model is consistent with the Klein et al. (2021) model of the process of explaining. Both of these models call out plausibility of transitions as a part of determining the stopping point.

In addition, our work may have useful methodological implications. Instead of studying fragmentary statements and static problems such as “Rebecca at the Beach” (her skin turned red or her skin turned turquoise) or “The bottle fell off the shelf. The bottle smashed” vs. “The bottle fell off the shelf. The bottle melted,” or scientific assertions, we examined naturalistic incidents that unfolded over time in complex settings. That methodology allowed us to discover the importance of story transitions, and the role of plausibility judgments in assessing these transitions. Future researchers may wish to build on this approach to expand the investigation of plausibility judgments. The methodology might include ways to code incidents to capture the trigger for story-building (anomaly/surprise, ignorance, etc.), leverage points for constructing the story, state transitions in the story, discrepancies in the state transitions, and strategies for repairing the story in order to reduce the plausibility gap.

The plausibility transition model may have implications for system design, particularly the design of advanced information technology involving automation, Artificial Intelligence and Machine Learning. Instead of treating these systems as opaque and inscrutable, designers may find it useful to offer system users transparency into the data used to train these systems so that the users can assess data quality in addressing plausibility gaps. Designers can make it easier for system users to track cognitive strain by flagging the discrepancies that emerge and highlighting the plausibility gaps.

With regard to training, developers can seek to make plausibility judgments more accurate by helping people build stronger mental models. (See the Mental Model Matrix developed by Borders et al., 2019, along with the PJCC model of Lombardi et al., 2016a). Developers can try to foster a mindset of curiosity regarding anomalies rather than dismissing anomalies as inconvenient. Developers can explore ways to encourage people to engage in counterfactual reasoning (as suggested by Kahneman and Miller, 1986) in identifying plausibility gaps and in assessing the quality of attempts to fill those gaps. Developers can seek methods to train perspective-taking skills to facilitate plausibility judgments involving other people. Developers can also try to help trainees gain a better appreciation of the limits of story-building in domains involving multiple intersecting causes.

Is the plausibility transition model testable? Here are some possibilities. (a) Individual differences will play a role in plausibility judgments. These include tolerance for uncertainty, curiosity, and need for closure; (b) Efforts to close a plausibility gap will diminish and cease as cognitive strain is reduced–not in a gradual way but abruptly as the state transitions click into place; (c) The detection of plausibility gaps will conform more closely to state transitions in a story than to factors such as probabilities and logical arguments.

## Data availability statement

The original contributions presented in the study are included in the article/Supplementary material, further inquiries can be directed to the corresponding author.

## Ethics statement

Written informed consent was obtained from the individual(s) for the publication of any potentially identifiable images or data included in this article.

## Author contributions

GK, developed the plausibility transition model, selected and reviewed the cases, wrote the initial draft, and completed the final draft of the manuscript. MJ conducted the literature review, prepared the analysis of the articles that were selected for further examination, and assisted in the editing of the manuscript. RH provided critiques and suggestions for the plausibility transition model, identified additional literature for inclusion in the manuscript, and made numerous editorial recommendations on the manuscript. SM provided valuable recommendations on the plausibility transition model from the very inception of the model. All authors contributed to the article and approved the submitted version.

## Funding

This research was developed with funding from the Defense Advanced Research Projects Agency (DARPA) under agreement number FA8650-17-2-7711. The United States Government is authorized to reproduce and distribute reprints for Governmental purposes notwithstanding any copyright notation thereon. The views and conclusions contained herein are those of the authors and should not be interpreted as necessarily representing the official policies or endorsements, either expressed or implied, of AFRL or the United States Government.

## Conflict of interest

GK and MJ were employed by MacroCognition, LLC.

The remaining authors declare that the research was conducted in the absence of any commercial or financial relationships that could be construed as a potential conflict of interest.

## Publisher’s note

All claims expressed in this article are solely those of the authors and do not necessarily represent those of their affiliated organizations, or those of the publisher, the editors and the reviewers. Any product that may be evaluated in this article, or claim that may be made by its manufacturer, is not guaranteed or endorsed by the publisher.

## Footnotes

^1^This interpretation of the pilot’s decision making is based on conversations with Nadine Sarter (personal communication), along with France’s Bureau of Enquiry and Analysis for Civil Aviation Safety (2012). Other accounts have been offered, such as the pilot’s inadequate training (Palmer, 2013).
