# Supplementary material for: The plausibility transition model for sensemaking
Source: Front Psychol. 2023 May 26;14:1160132. doi: 10.3389/fpsyg.2023.1160132 (PMC10251660; doi:10.3389/fpsyg.2023.1160132)
Supplement: Supplementary file 1 [file Data_Sheet_1.pdf]

Appendix A

Listing of the Cases

These cases are ones discovered in literatures or the media that were particularly informative of the Plausibility Gap Model. Links to the sources can be provided on request.

Cases Involving Machines or Information Technology

1. Why did Watson give the answer "Toronto" in Jeopardy?
2. Why are there maggots in my dead refrigerator?
3. Why did Air France #447 crash?
4. How does AlphaGo work?
5. Distillation tower: why did operators miss the upset in Scenario 3?
6. Why did my GPS take me down an absurd route?
7. Why did CPT Rogers of Vincennes shoot down a commercial airliner in 1988?
8. Why did KAL 007 get shot down?
9. Why did the cruise ship Royal Majesty get grounded?
10. Why did the airplane crash after the pilot failed to arm the spoilers?
11. Why did the automatic blood pressure machine fool the surgical team?
12. Why do autopilots sometimes quit working with no warning?
13. In Desert Storm, why did our Patriot missile system shoot two friendly airplanes?
14. How do trains negotiate curved tracks?
15. How did the firefighter know to order his crew out of the burning building?
16. Why did the British naval officer order the shootdown of a new track?
17. What caused the mysterious outages on the Singapore subway system in July 2017?

Cases Involving other Complex States of Affairs, Events, or Circumstances

18. Why did Walter Reed believe that mosquitoes were not involved in spreading Yellow Fever when he went to Cuba to study the disease?
19. Why did the police officer shoot the innocent African American shopper at the WalMart in Beavercreek, OH?
20. Why did the Department of Justice confrontation with Koresh end in disaster?
21. Why do Westerners and Arabs baffle each other in the way they think?
22. Why was the German Blitzkrieg successful against the French in WW2, even though France had a very strong military?
23. Why did the US Navy ship John S. McCain collide with another ship near Singapore in 2017?
